# RIVETS: A Mechanical System for In Vivo and In Vitro Electrophysiology and Imaging

**DOI:** 10.1371/journal.pone.0089007

**Published:** 2014-02-14

**Authors:** Jason E. Osborne, Joshua T. Dudman

**Affiliations:** Howard Hughes Medical Institute, Janelia Farm Research Campus, Ashburn, Virginia, United States of America; Baylor College of Medicine, United States of America

## Abstract

A number of recent studies have provided compelling demonstrations that both mice and rats can be trained to perform a variety of behavioral tasks while restrained by mechanical elements mounted to the skull. The independent development of this technique by a number of laboratories has led to diverse solutions. We found that these solutions often used expensive materials and impeded future development and modification in the absence of engineering support. In order to address these issues, here we report on the development of a flexible single hardware design for electrophysiology and imaging both in brain tissue in vitro. Our hardware facilitates the rapid conversion of a single preparation between physiology and imaging system and the conversion of a given system between preparations. In addition, our use of rapid prototyping machines (“3D printers”) allows for the deployment of new designs within a day. Here, we present specifications for design and manufacturing as well as some data from our lab demonstrating the suitability of the design for physiology in behaving animals and imaging *in vitro* and *in vivo*.

## Introduction

A typical adult laboratory mouse used for experiments in neuroscience weighs approximately 30 grams. This modest body weight and size allows a typical mouse to carry devices for electrophysiological or imaging measurements of approximately 3 grams (often with counterweighting) while still performing freely moving behavioral tasks. While impressive efforts have for many years sought to miniaturize recording devices under these constraints in mice and other small animals, it is clear that substantial engineering effort can be avoided by using restrained, behaving mice. Head-fixed experiments in awake, behaving mice and rats have opened up the possibility for robust, high signal to noise imaging experiments [1]–[4] combined with both intracellular [5]–[10] and extracellular [11]–[16] electrophysiology with large, stable optical and electrophysiological equipment.

The development of behavioral and electrophysiological analysis of single neurons in behaving, head-fixed primates was pioneered in the 1960’s [17], [18] and built upon earlier work that developed and refined methods for extracellular recording of single neurons (*e.g.* [19], [20]). The core feature of the approach was to mechanically fix a hard plastic chamber to the skull centered over a craniotomy. This approach allowed reliable access for acute recordings over periods of many months while allowing awake subjects to learn and behave tasks comfortably. The equipment necessary to replicate these experiments in primates is now commercially available from a number of sources (*e.g.* http://www.artcorp.com). The techniques have been adapted to target recordings to a large number of cortical, subcortical, and peripheral targets in the primate nervous system.

As multiple laboratories have developed an interest in the use of head-fixed, behaving rodents for electrophysiology and imaging a number of methods have been adapted to the needs of individual laboratories, but a general solution does not yet exist. Thus, we sought to develop a general approach suited to a wide array of experiments that optimized several features: (1) Sufficient stability for electrophysiology and imaging; (2) Minimized experimental cost; (3) A flexible design that facilitated customization according to experimental needs; (4) Capacity to be produced and shared amongst groups easily. Here, we describe a design that we believe optimizes across these constraints and is likely to be of use to other laboratories working with mice and other small animal preparations. Furthermore, the design files, together with detailed advice for their manufacture, and hopefully future designs contributed by users will be made available through the web (http://dudmanlab.org/html/rivets.html).

## Results

### Design and Implementation

Decades of work in awake, behaving primates have demonstrated that hard plastics such as Delrin are sufficient for stable acute recordings via a chronically implanted chamber. By contrast, many laboratories working with mice and other small rodents have generally developed head-fixation chambers that are machined out of very hard metals such as titanium. The use of a plastic chamber produces a number of benefits over metal chambers including a greatly reduced cost, an increased flexibility in design, and an improved capacity for production. We further reasoned that the most significant gains could be achieved if we could exploit a 3D rapid prototyping machine for the production of recording chambers. For ease of reference later in the text we named the chamber design: rodent in vitro/vivo electrophysiology targeting system or RIVETS™.

**Figure 1:**
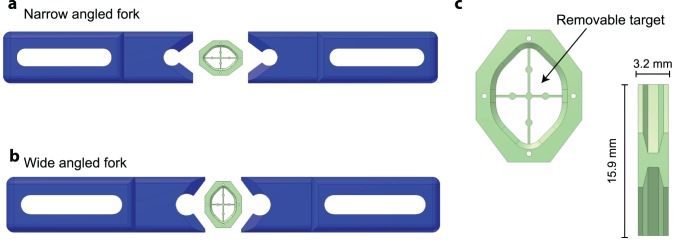
RIVETS Head post and locking forks. (a) RIVETS head post with removable target (b) RIVETS head post shown with narrow forks for implants in the x axes (c) RIVETS head post shown with wide forks for implants in the y axes.

The initial chamber design followed from a simple concept: a chamber needed to provide central access for physiology while providing an outer surface sufficient for stable clamping and holding of position. We reasoned that optimal stability could be achieved using a set of surfaces with 45° angles between surfaces. A set of surfaces on each side of the chamber provides robust holding force directed along all 3 dimensions. A number of plastics and designs were tried initially (not shown), however, we have commonly manufactured RIVETS chambers using two types of 3D printer resin, VeroClear FullCure810 and VeroWhitePlus FullCure835 with a flexural strength of 75–110 Megapascal (MPa) tensile strength 50–65 MPa and print layer resolution of 16 µm using an Objet Connex 350 3D rapid prototype printer (Figure 1). The majority of data on stability and recording experiments were conducted with chambers produced from this material, although other materials likewise produced successful results [16].

The longest lever arm in the proposed design is the extendable clamps (Figure 1). To ensure stability of the clamping system we manufactured the clamps and associated elements out of 316 Stainless Steel. The RIVETS fork system (Figure 1b,c) has a matching angle configuration locking method which restrains the head post by applying pressure in x,y and z directions when forced together. Using the narrow or wide forks, the researcher can mount the head post to favor x or y implants.

Together this design allowed us to manufacture a number of chambers at low cost without need of recovery of individual chambers. Relatively simple surgical procedures (see Methods) for chronic attachment of chambers have reliably allowed our lab to assess head-fixed behavior and learning in individual mice for many months. One early concern was the repeatability of the mounting of chambers to the head. We addressed this concern with a design feature that was simple to implement using a rapid prototype printer. A thin, removable targeting system is printed in the center of the chamber to allow the chamber to be reliably positioned either centered at bregma or relative to a landmark such as bregma (Figure 1).

Below we detail our measurements of the stability of the device as applied to a number of example experiments from our lab.

The pRIVET provides stability in a chronic head-fixed behaving preparation.

The initial design we implemented and tested we refer to as the physiology RIVET (pRIVET). It is a relatively low profile chamber (∼3 mm height) that provides access to a large fraction of the dorsal surface of the mouse brain (a ∼5×10 mm area) and can be used for recordings from a number of cortical and subcortical structures. Chambers were chronically attached to the skull (described in Methods) of 5 adult mice (∼30 g). Following recovery from surgery mice were trained to press a lever for a sweetened water reward (behavioral paradigm to be described elsewhere).

**Figure 2:**
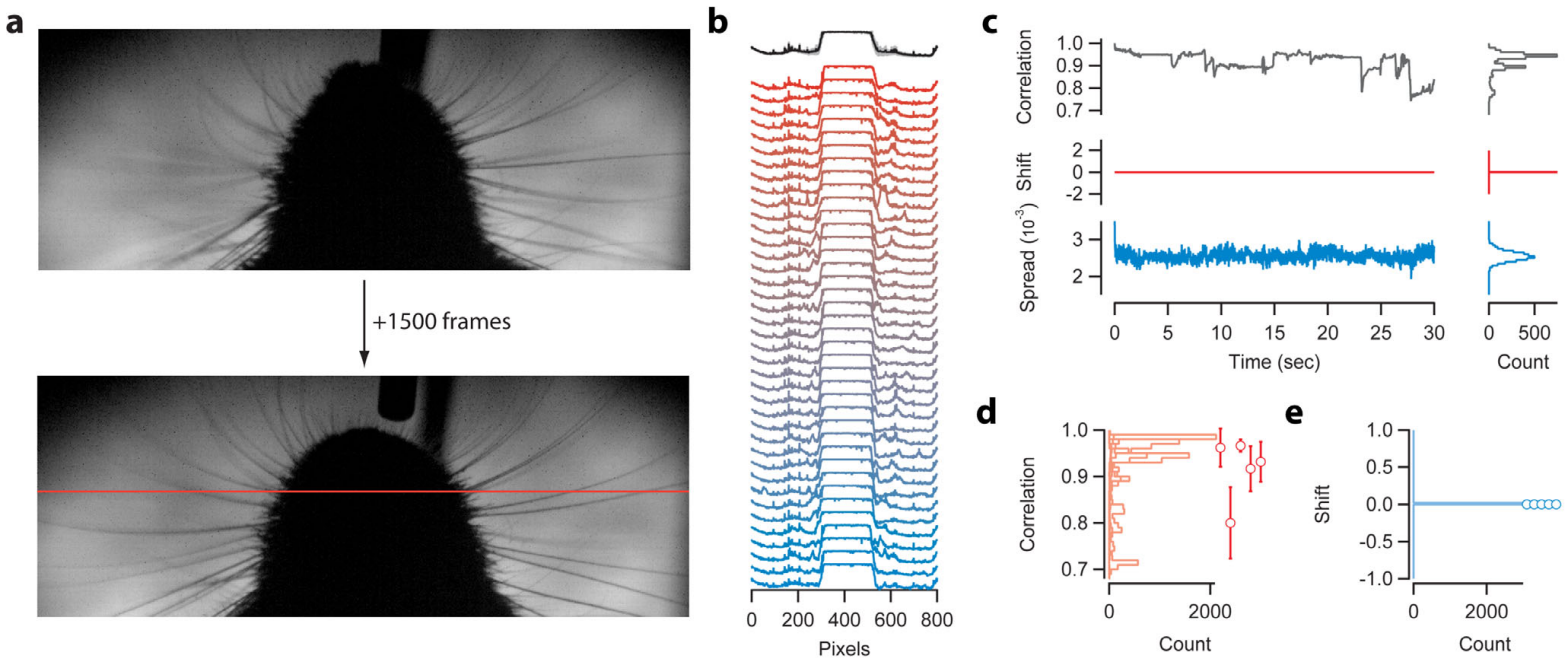
Stability of RIVETS in awake, head-fixed mice. (a) Two example images from a behavioral session with a mouse implanted with the RIVETs chamber. Video was collected at 100 Hz. (b) For each frame a line profile of intensity (inverted) was measured. Upper trace shows the mean (black) and standard deviation (gray) from line profiles collected for 3000 frames of a behavioral session. Example profiles spaced every 50 frames from early (red) to late (blue) profiles are shown. (c) From individual image profiles the correlation (upper), shift in the maximal location of cross correlation (middle), and the fractional difference between max correlation value and next greatest correlation score (lower) are shown for an example session with a single mouse. (d) Quantification of the profile correlations are shown for 5 mice in 5 sessions. Both histograms for inidivdual mice (light red) and the mean +/− standard deviation (red circles) are shown. (e) In all 5 mice no apparent shift was detected between frames as shown by the individual histograms (light blue) and means (blue circles).

We first sought to assess the stability of the pRIVET and clamping system by measuring the displacement of the head of a trained mouse during performance of the operant task. A CMOS camera was positioned above the head and snout of the mouse at an angle approximately 30° from vertical. Video was recorded at 100 frames/sec for 15 minutes of task performance (typically many tens of trials). Example video frames from the beginning and end of a typical video recording session are shown in Figure 2a. To assess stability we measured the correlation of both individual video frames and the spatial profile across the approximate center of the nose of the mouse (Figure 2b,c). The frame to frame correlation relative to the initial frame was calculated to measure the amount of movement (change in pixel intensities) in the image. By contrast we used the framewise cross-correlation to determine whether the main object in the image (the snout of the mouse) was displaced. Displacement would be reflected by a non-zero shift in the peak of the cross correlation. Despite the fact that there was substantial movement across frames as indicated by correlation scores distributed below 1 across each of the 5 mice used (Figure 2c,d) we found that the snout of the mouse did not shift as indicated by a peak in the cross correlation function at a zero pixel offset (Figure 2c,e). Thus, for the 5 mice tested we found that despite substantial movement of the face and whisker pad during the lever pressing task (correlation scores less than 1; Figure 2d) the maximal alignment position of the image was never observed to shift (Figure 2e).

The pRIVET provides sufficient stability for extracellular recording in awake mice.

**Figure 3:**
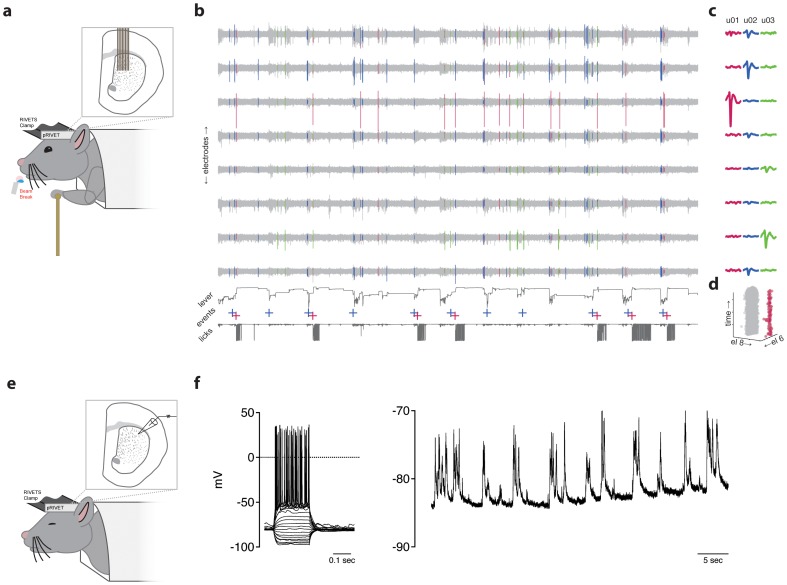
In vivo electrophysiology using RIVETS. (a) Schematic of the recording configuration used to obtain recordings shown in b–d. Briefly, a mouse trained to pull a lever (brass color) for water reward (blue water drop) was fixated in the RIVETS system under anesthesia. A small craniotomy was made and an 8 shank silicon probe array with 64 recording sites was lowered in to the dorsal striatum (depicted in the inset). Recordings were initiated ∼1 hour after recovery from anesthesia when the mouse was awake and actively moving the lever to obtain a water reward. (b) 2 minutes of a continuous recording from a single shank of an 8 shank silicon probe array is shown (‘electrodes’, light gray traces). Sorted spikes were colored according to the cluster to which they belonged and are indicated in overlay on the recording traces. Simultaneous monitoring of the lever position and beam breaks (‘licks’) are shown in the lower traces. Two types of events were detected and recorded in real time: threshold displacement of the lever (blue crosses) and delivery of water reward (red crosses). Rewards were delivered on a random subset (∼75%) of threshold crossings. (c) Averaged waveforms for the duration of the recording shown in b and separated by cluster (u01–u03) are shown for a window of −1 to +2 ms around the detected event. (d) The amplitude of each event in cluster u01 is projected into the space of electrodes 8 and 6 (el8 and el6) as a function of time (z-axis) for the entire 18 minutes of recording in this particular session. (e) Schematic of the recording configuration used to obtain recordings shown in f. As in the experiment depicted in a, a mouse was anesthetized and a small craniotomy was performed. The mouse was left lightly anesthetized while striatal neurons (∼2.5 mm below surface) were targeted from whole-cell patch clamping. (f) Representative whole-cell recording from a putative medium spiny neuron in the dorsal striatum. Left plot shows the response to a family of current steps from −160 to +160 pA in increments of 20 pA. Right plot shows 50 seconds of continuous recording showing a hyperpolarized resting potential and repetitive periods of sustained excitatory input.

Our video analysis of awake, behaving mice suggested that the pRIVET would be sufficiently stable for extracellular recording. We have now performed a number of recording experiments of which we provide examples in Figure 3. A schematic of the preparation for head-fixed operant conditioning is presented in Figure 3a. Electrode arrays can then be acutely implanted into brain structures of interest. In the example provided we implanted a 64 channel silicon probe array into the dorsal striatum of a trained mouse (previously used for video analysis, Fig. 2).

The quality of extracellular recording data is determined largely by the stability of the extracellular waveform. This is required for reliable spike sorting over time in the recording session. We show a continuous 120 seconds of recording (voltage signals were bandpass filtered from 300–7000 Hz and sampled at 30 kHz) from one shank of the 64 channel array (Figure 3b). Time aligned behavioral data is shown in the lower plots including the movement of the operant lever (‘lever’), times of reward delivery (red crosses), and the collection of reward from the water spout (‘licks’). Spike sorting allowed the ready identification of easily separable units, of which 3 examples (u01–u03) are shown (Figure 3c). Units were stable despite robust behavior from the mouse. For the entire 20 minute recording session we show one projection to demonstrate the robust stability of u01 throughout the recording (Figure 3d). These data are representative of the recording quality and stability that can be obtained with the pRIVET recording chamber in a well-trained mouse.

Using the recording techniques described above we found excellent stability of single units in behaving mice. For example, we have recently collected a dataset from which we isolated 396 single units. From this population of cells we found that that the mean duration of stable isolation was 30.1±14.7 (s.d.) minutes with a maximum duration of 69.0 minutes. These population numbers underestimate the number of unstable units that were not sorted and likewise under estimate the total possible duration of recording since many sessions were terminated despite continued, stable unit isolation (generally because sufficient behavioral data had been collected). Thus, these numbers may be thought of as a reasonable expectation of performance for silicon probe array recordings in head-fixed mice performing an operant task. This is comparable to the reported duration of extracellular recordings (“∼15–30 minutes”) in mice head-fixed with metal bars as reported previously [8].

The pRIVET provides sufficient stability for intracellular recording in anesthetized mice.

To further assess the mechanical stability of the pRIVET chamber we also performed whole-cell patch clamp recordings from neurons in the dorsal striatum of lightly anesthetized mice (schematized in Figure 3e). Whole-cell recordings are very susceptible to motion artifact and thus provided a good test of the mechanical stability of the holding system. We were readily able to obtain whole-cell recordings from neurons in deep structures such as the dorsal striatum. An example of one such recording is shown in Figure 3f. Good access and stable recordings permitted measurement of spiking responses following a family of current injection steps (Figure 3f, left) and long, continuous recordings revealed the sequence of up-state to down-state transitions (Figure 3f, right) typical of striatal neurons in lightly anesthetized animals [10], [21]. For a sample of 5 whole cell recordings performed using the pRIVET under these conditions we collected data from stable whole cell recordings from 16.4±6.3 minutes (s.e.m.) with one recording lasting ∼37 minutes. These values again represent a likely underestimate of maximal recording time as some recordings were terminated prior to losing the cell; however, they may reflect typical or expected performance as assayed in this small sample.

### A Common System with Multiple Chamber Designs for Multiple Preparations

**Figure 4:**
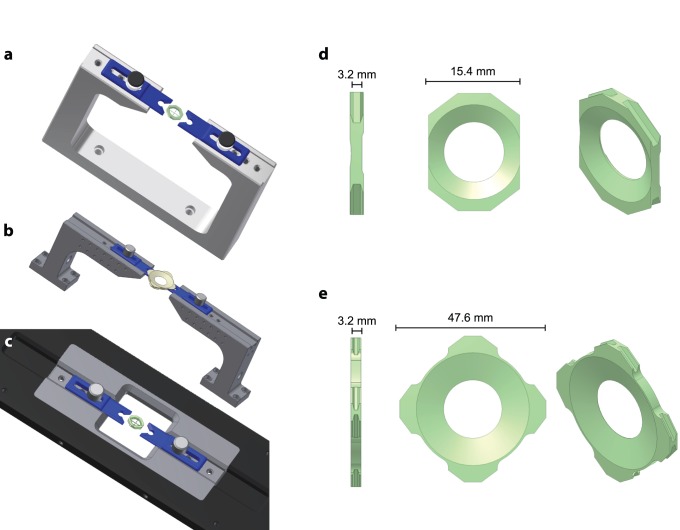
Configurable stereotaxis, head post and slice chamber. (a) RIVETS shown with simple one piece stereotaxis (b) RIVETS with customized two piece stereotaxis system (c) RIVETS with integrated stereotaxis insert for xy stage platform (d) RIVETS low profile optical head post (e) RIVETS *in vitro* slice chamber.

For a number of laboratories in vitro experimentation in acute brain slices is combined with physiology and imaging experiments in vivo. To facilitate the combination of these techniques on a single piece of equipment (*e.g.* a two-photon microscope) we have designed and fabricated a number of modified RIVET systems (Figure 4). In more recent versions of the system the holding systems for clamping forks were fabricated from 420c stainless steel and heat-treated to 50 Rockwell (Rc) hardness for stability and wear resistance. A simple single piece fork holder stereotaxis (Figure 4a) can be mounted to an air table or breadboard while allowing clearance for a head fixed animal. If more flexibility is desired (Figure 4a), the stereotaxis is customized with two independent fork holders allowing adequate clearance for imaging or head fixed behavior systems. If translation of the stereotaxis is required the RIVETS can be customized into an insert configuration and mounted into a xy stage platform (Figure 4c). Imaging and acute procedures using the RIVETS system are made possible with a low profile head post (oRIVET; Figure 4d).

**Figure 5:**
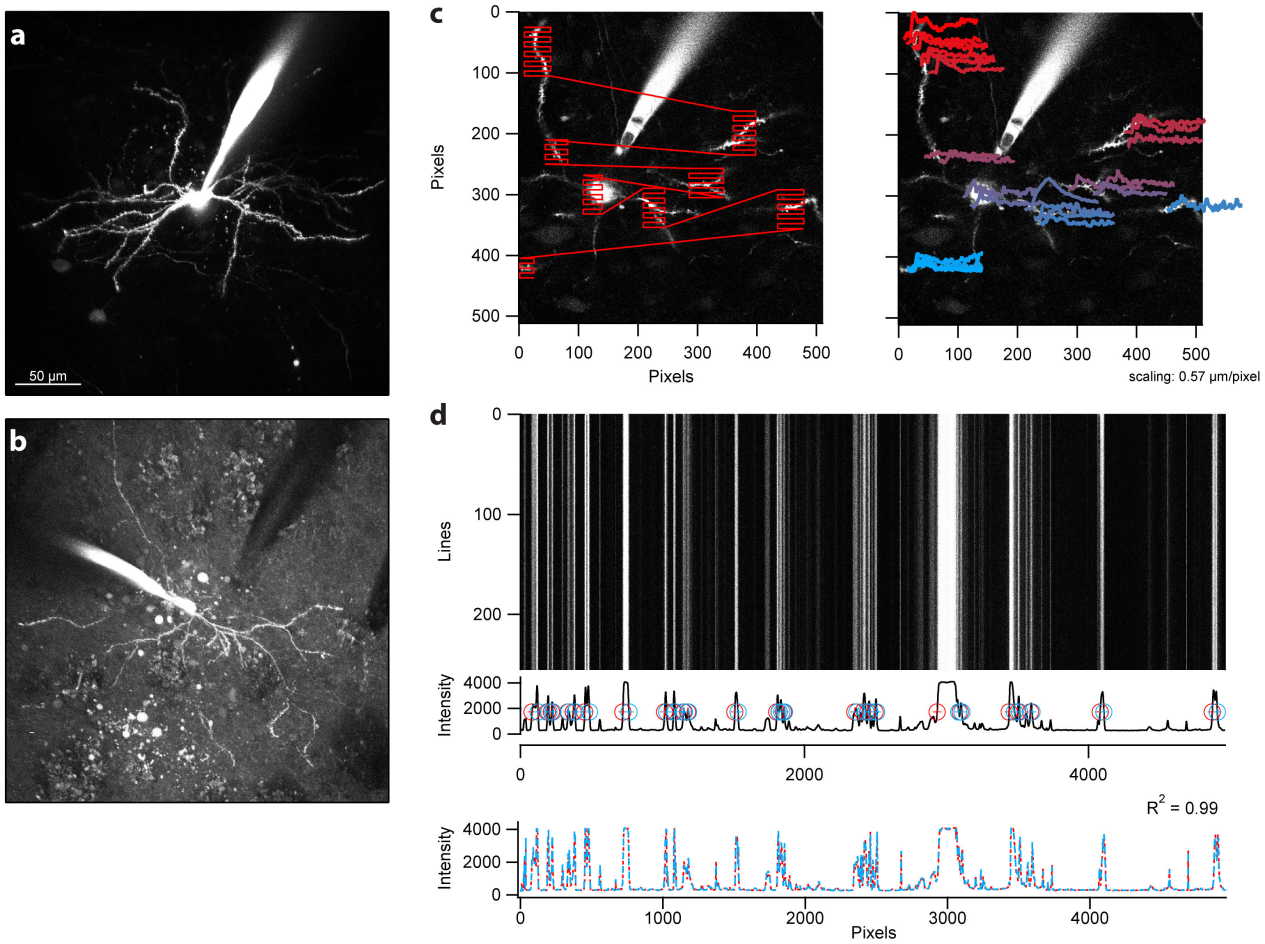
In vitro patch clamp electrophysiology and subcellular imaging using RIVETS. To assess the stability of the slice chamber configuration we provide an example of 2 of the more challenging applications for in(a–b) First, we show that simultaneous whole-cell recordings from multiple nearby neurons can readily be obtained. In this case each neuron was filled with either a red (a; Alex594) or green (b; Alexa488) dye through the recording pipette. We further show that recorded neurons are sufficiently stable to perform detailed calcium-imaging experiments from the dendritic arbor. (c) A neuron was filled with both a red (Alexa594) dye and a calcium-sensitive fluorescent dye (Fluo-8). A complex linescan pattern covering multiple locations within the dendritic arbor was designed (left image, red overlay). The resulting calcium transient evoked by a barrage of backpropagating action potentials in shown in the colored traces at right. The colorization reflects progress in the linescan from beginning (cyan) to end (red). (d) To confirm the stability over many linescans the obtained structural image (red channel) was aligned for each line for a sequence of 250 lines. The maximum intensity trace and detected peaks for the image is shown in the middle plot. Red and blue crosses indicate the left and right edges of each detected structure, respectively. In the bottom plot we overlay the maximum intensity projection along the line for two sequences of line scans taken several minutes apart. The R^2^ value of the correlation between these two maximum intensity projections was 0.99. Units of intensity are arbitrary.

To allow a single microscope to be rapidly interchangeable between acute experiments in brain slices and in vivo experiments we designed and fabricated a low profile slice chamber (sRIVET; Figure 4e). The sRIVET has a recessed underside for a mounted 22 mm×22 mm cover slip. We have used this system for acute electrophysiology and imaging. The chamber can also be rapidly exchanged either between in vitro experiments or between preparations. Although a number of plastic slice chambers are available we nonetheless sought to ensure that the clamping system used in the sRIVET design provided the necessary stability of typical in vitro experiments. To test stability we have examined the application most critically dependent upon stability in our lab: two-photon calcium imaging of subcellular compartments. Imaging of individual dendritic segments in neurons in acute brain slices depends upon the stable position of recorded neurons over the time course of tens of seconds to minutes. In an example experiment, we assessed stability of cell position by designing a fast linescan pattern that crossed the dendrite of a filled neuron several times per frame (Figure 5a). This allowed us to examine both the stability of the neuron across repeated linescans (∼1 ms interval; Figure 5c) and the stability of a particular linescan pattern for scans separated by ∼30 seconds (Figure 5d). The stability of the sRIVET allowed us to measure clear calcium transients from multiple postsynaptic compartments (Figure 5b). We find that these results are typical for a large number of standard in vitro experiments performed in our laboratories.

### A Modified Design for in vivo Two-photon Imaging

**Figure 6:**
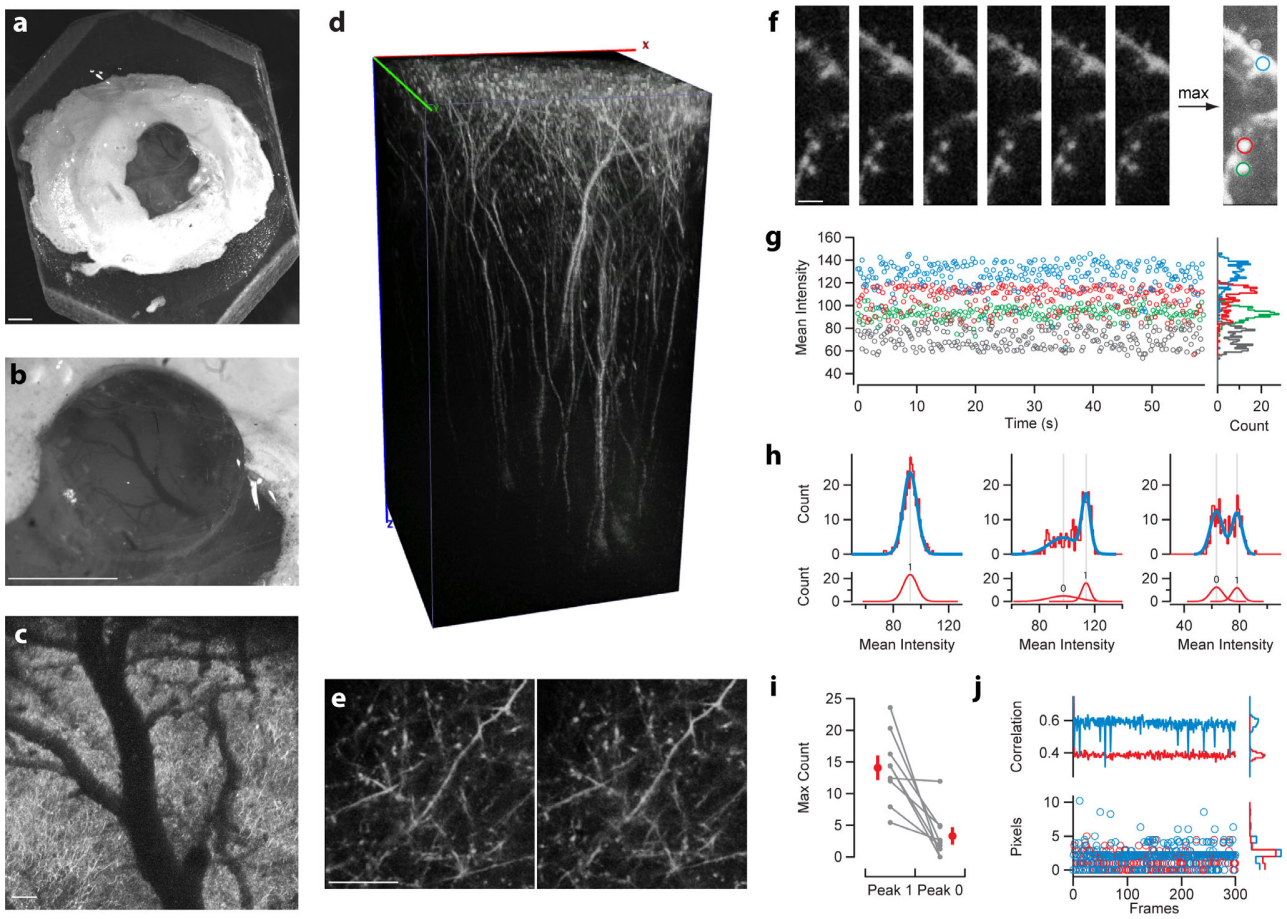
In vivo two-photon imaging using RIVETS. (a–c) A custom designed RIVETS holder was made to facilitate access of microscope objective (a). A craniotomy of ∼1.8 mm diameter was made and a small glass coverslip placed in the craniotomy (a–b). Imaging began by taking a low power (10×, 0.3 NA) of the surface of the brain (c). Upper image in (d) shows a volume reconstructed using a 60× (0.95 NA) objective to image layer V pyramidal neurons to ∼800 microns below the pial surface. Lower images show a single imaging plane collected 10 minutes apart. To assess stability of RIVETS small regions were imaged at 4–5 Hz. Single images taken 15 seconds apart (e) and the maximum intensity projection of the complete 300 frame video. To quantify stability both small ROIs (f–h) and whole frame correlations (i) were calculated. Z-axis displacement was estimated as changes in intensity of fluorescence of small ROIs. 4 example ROIs are shown in f over time. Histograms of all intensity values are plotted on right inset. Intensity histograms exhibited several forms ranging from highly consistent values through fluctuations in intensity (g). The distribution in peak heights of a multipeak histogram fits (h) reveal that most ROIs are stable across frames with a single dominant peak. Lateral shift can be estimated from the correlation between frames (i, upper plot) and the distance of the peak of the normalized cross-correlation (i, lower plot). Scale bars: a–b, 1 mm; c, 100 µm; e, 25 µm; f, 2.5 µm.

One feature of the RIVETs design is the ability to rapidly design, fabricate, and utilize a new chamber design suited to particular experimental needs. Above we demonstrated that modifications of the RIVET system could be used to perform extracellular recordings from head-fixed mice as well as two-photon calcium imaging in acute brain slices. We next sought to modify the RIVET design to produce a chamber suitable for in vivo two-photon structural imaging. We chronically implanted an oRIVET chamber (Figure 4d) onto the skull of 2 Thy1-YFP mice (Figure 6a). A small (∼2 mm) craniotomy was made through the skull, filled with low density agarose and covered with a small glass coverslip (Figure 6a–b). Image stacks at both low magnification (Figure 6c) and high magnification (Figure 6d) revealed high resolution and low jitter consistent with stable imaging in a quietly resting animal that had been habituated to the restraint system. We found that full field frames were very stable over minutes (Figure 6e). Finally, to assess stability we evaluated the displacement of individual image frames acquired at typical imaging rates (∼4 frames/sec; 75 seconds of imaging which is more than a typical trial in a behavioral task; Figure 6f–j). To quantify the stability of the image in the z and xy planes we computed intensity histograms of small ROIs (Figure 6g–h) and frame-by-frame cross-correlations (Figure 6i–j), respectively. Z stability is indicated by changes in intensity that reflect the movement of small objects in and out of the plane of focus. Such fluctuations should be reflected by a bimodal structure in the intensity histogram such that on some frames the object is out of focus and emits few emission photons whereas in other frames the object is in the focal plane and emits relatively many photons. Indeed we found that small ROIs could exhibit such bimodal distributions (Figure 6g,h). First, we reasoned that z stability could be estimated as the fraction of frames in which a small object was found in the current image plane by comparison to the number of frames in which the object was out of the plane of focus. To estimate the fraction of frames containing the object in focus we fit the histograms as a mixture of two Gaussian distributions (Figure 6h). The relative amplitude of these Gaussian distributions were generally strongly biased towards frames in which the object remained in focus and in some cases ROIs could be extremely stable with essentially no frames in which the object was out of focus (Figure 6i). Given the existence of some highly stable objects this suggests that globaly the position of the brain in z was very stable with local fluctuations of small objects (generally dendritic spines) object a stable central position. Moreover, drift would have been revealed by a systematic bias towards low intensity which was not observed (Figure 6f,g). Finally, we sought to assess the stability of the image in the xy plane. Similar to methods used to correct for image movement in the xy plane we first calculated the frame-by-frame cross correlation and found the distance to the peak of the cross correlation function. Using this techniques we found that in general images were stable in xy position with the vast majority of shifts being less that 3 pixels with occasional larger shifts of up to 10 pixels (Figure 6j). This is comparable to the magnitude of shifts corrected by existing techniques [4], [22].

## Discussion

It has become relatively common practice in many neuroscience research laboratories to perform experiments using electrophysiology and imaging approaches applied both in vitro and in vivo in mice. The significant expense of equipment for high channel count electrophysiology recordings and two-photon imaging microscopes place a premium on the efficient combination of equipment for multiple uses. Moreover, the increasing interest in performing experiments in head-fixed, behaving rodents, especially mice, demands effective and convenient mechanical solutions. Finally, the speed at which experimental designs change and the great variance in techniques applied across laboratories can make general solutions difficult.

We have developed a system, RIVETS, that provides a flexible platform for experimentation. Our solution attempts to optimize across a number of features including the ability to rapidly convert equipment between multiple experimental approaches, rapid design and customization for new experiments or techniques, low cost and ease of production, all while not sacrificing stability and recording quality. Despite the low cost and ease of production we find that multiple available materials provide excellent stability for a range of experiments from extracellular recording to subcellular imaging. We show for example stable positioning of the head of a trained mouse performing an operant task (Fig. 2). The system is suitable for extracellular recording from behaving mice (Fig. 3) and imaging in quietly resting mice (Fig. 6) and, with image correction routines, from behaving mice. The flexibility of the design has also made it possible to rapidly convert between a rig suitable for *in vivo* (Fig. 3) and *in vitro* (Fig. 5) intracellular recordings.

As in the design industry, our design achieves flexibility of implementation by leveraging rapid prototyping machines (3D printers) for the purpose of cutting-edge neuroscience experiments. With the burgeoning availability of low cost, desktop 3D printers and a number of commercial suppliers of inexpensive 3D printed materials we believe that RIVETS will find application broadly in the neuroscience community. We have many examples of the rapid modification of this design for a new experiment performed in our lab. RIVETS are currently in use by several laboratories at the Janelia Farm Research Campus and our intent with this publication is to make this system broadly accessible to neuroscience laboratories at other institutions. Additional work can be required to incorporate the RIVETS design and concept into existing microscope and physiology rigs; however, we find that the flexible design allows this to be easily achieved and provide some examples from our own laboratories. The necessary information, files, and manufacturing details for producing a customized RIVETS system can be obtained from our website: www.dudmanlab.org/html/rivets.html.

## Methods

### Fabrication of RIVETS

Full fabrication methods, potential suppliers, and downloadable CAD files will all be made available online (www.dudmanlab.org/html/rivets.html). Briefly, all components were designed in CAD software (www.autodesk.com). We have commonly manufactured RIVETS chambers using two types of 3D printer resin, VeroClear FullCure810 and VeroWhitePlus FullCure835 with a flexural strength of 75–110 Megapascal (MPa) tensile strength 50–65 MPa and print layer resolution of 16 um. Earlier fabrications were performed with a 720 full cure PolyJet resin with a flexural strength of 75.8 Megapascal (MPa), tensile strength 60.3 MPa with comparable performance. All printing was done using a Objet Connex 350 3D rapid prototype printer (www.stratasys.com/3d-printers/design-series/precision/objet-connex350). In our experience the currently available desktop 3d printers (e.g. MakerBot) were not able to achieve the necessary resolution to obtain working RIVETS systems, however, these printers are rapidly improving and thus we are hopeful that future designs will be capable of supporting applications like RIVETS. The sliding clamps and arms that support the RIVETS system have typically been machined from 316 Stainless Steel.

On the associated website (www.dudmanlab.org/html/rivets.html) we provide links to information necessary for fabrication through outside vendors. For example, we provide an example of an external vendor that could supply the necessary printed RIVETS (http://www.artcorp.com) using the CAD drawings that we are making available. Finally, we have provided a link to the Tool & Die Company with whom we have frequently outsourced the production of the forks and clamping apparatus (http://ronal.com). We have now been certain to put all of these links in the methods section. In most cases it is not possible to provide estimates of cost given their sensitivity to the number of devices that are ordered. However, in our experience the cost of individual RIVETS head chamber is less than $0.20 per RIVET on our inhouse 3D printer. We did receive quotes from outside print houses with prices around $5 a piece with a 100 piece order. The cost of the fork and clamping system is comparable to the cost of a stereotaxis ($4–5 k). However, we have found that for many experiments simple solutions using combinations of parts can be used in conjunction with precisely machined forks and thus obtained at substantially lower cost.

### Ethics Statement

All animals were handled in strict accordance with guidelines approved by the Institutional Animal Care and Use Committee (IACUC) of the Janelia Farm Research Campus. This project was approved by IACUC and conducted under protocols 8–36 and 11–69.

### Subjects and Training

For behavior and *in vivo* experiments we report results from 7 adult (30 g; 3–6 months old) male mice (C57/Black6) from the in house breeding colony. Mice were initially housed in a temperature- and humidity-controlled room maintained on a reversed 12 h light/dark cycle. Following one week of recovery from surgery to implant the RIVETS head cap, the water consumption of the mice was limited to 1.5 mL per day primarily acquired in the head-fixation apparatus. Mice under went daily health checks, and water restriction was eased if mice fell below 75% of their body weight at the beginning of deprivation. Mice were then familiarized with the head-restraint apparatus which was located inside a sound and light attenuating enclosure. Mice were trained to obtain fluid from a water spout positioned a few millimeters in front of their mouth. Small volumes (≈0.01 ml) of water were delivered to the spout via a computer control using custom software (written by J.T.D.) and electronics with a nominal time resolution of 1 kHz (to be described elsewhere). Licks of the water spout were detected using an infrared beam break. Displacement of a lever located near the front forepaw of the mouse produced a delayed water reward on a randomly assigned subset of trials (∼85%). Mice rapidly learned to press the lever for reward. Data obtained in figures 2 and 3 were taken from mice that had undergone at least 2 weeks of operant training. Data in figure 6 was obtained from a mouse that had been habituated to head fixation, but was not performing an operant task.

### Affixing the RIVETS Chamber

To affix the chamber to the skull we adapted published techniques [23]. Briefly, mice were deeply anesthetized (isoflurane; 1.5–2%) until all reflexes were suppressed and a slow, steady rate of respiration was obtained. A bolus injection of local anesthetic was provided subcutaneously over the skull. An incision was made longitudinally and the skin was pushed to the side to reveal the dorsal surface of the skull. The surface of the skull was cleaned and dried extensively under visual inspection. The surface was roughened to provide a surface for affixing the chamber. A chamber was then affixed to the skull using dental cement. Due to the rather large size of some chambers (e.g. oRIVET) a more viscous mixture of dental cement was prepared so as to build “bridges” of cement out from the skull to the lateral portions of the chamber. This technique is similar to that used to build up towards elevated components of chronically implanted electrode arrays, for example. Mice were carefully monitored and given post-operative analgesics for 3 days following surgery. For a subset of experiment (Fig. 3 and 6) a craniotomy was required prior to experimentation. Briefly, a trephine shaped bit of 1.5–2 mm diameter was used to remove a small circular piece of skull over the cortex. The installation of the cranial window for imaging is described below. For electrophysiological experiments the dura mater was carefully removed using a 30 gauge needle and #3 forceps. Following dura removal electrodes were slowly lowered into the brain under visual guidance. Care was taken to avoid vasculature during implantation of electrodes. Subsequent steps are described below.

### In vivo Electrophysiology and Imaging

Following recovery, habituation, and training a small craniotomy was made under deep anesthesia and electrode arrays were maintained in position by a micromanipulator (Scientifica). The silicon probe array (NeuroNexus Technology) was slowly lowered in to the dorsal striatum (coordinates relative to bregma medial: 1–3 mm; anterior: 0.5 mm; ventral: −2.5 mm). After >1 hour of recovery recording data was obtained from alert, behaving mice previously trained on the operant task. Broadband (0.1 Hz-7 kHz) data was sampled at 30 kHz from all 64 channels and acquired synchronously with digital and analog signals for behavioral measurements (Cerebus system; Blackrock Microsystems). Zero-phase filtering, spike detection, bandpass filtering, and clustering were performed offline using custom written Matlab programs (written by J.T.D.).

### Intracellular Recording and Imaging

Briefly, mice were deeply anaesthetized under isoflurane, decapitated and the brains were dissected out into ice-cold modified artificial cerebral spinal fluid (aCSF) (in mM: 52.5 NaCl, 100 Sucrose, 26 NaHCO_3_, 25 Glucose, 2.5 KCl, 1.25 NaH_2_PO_4_, 1 CaCl_2_, 5 MgCl_2_ and in uM: 100 Kynurenic Acid) that had been saturated with 95%O_2_/5%CO_2_. 300 µM thick coronal slices were cut (Leica VT1200S; Leica Microsystems, Germany), transferred to a holding chamber and incubated at 35°C for 30 minutes in modified aCSF (in mM: 119 NaCl, 25 NaHCO_3_, 28 Glucose, 2.5 KCl, 1.25 NaH_2_PO_4_, 1.4 CaCl_2_, 1 MgCl_2_, 3 Na Pyruvate and in uM: 400 Ascorbate and 100 Kynurenic Acid, saturated with 95%O_2_/5%CO_2_) and then stored at room temperature.

For recordings, slices were transferred to a recordings chamber perfused with modified aCSF (in mM: 119 NaCl, 25 NaHCO_3_, 18 Glucose, 2.5 KCl, 1.25 NaH_2_PO_4_, 1.4 CaCl_2_, 1 MgCl_2_, 3 Na Pyruvate and saturated with 95%O_2_/5%CO_2_) maintained at 32–34°C, at a flow rate of 2–3 mL per minute. Patch pipettes (resistance 5–8 MΩ) were pulled on a laser micropipette puller (Model P-2000, Sutter Instrument Co., Sunnyvale,CA) and filled with one of the following intracellular solutions: Current-clamp recordings of spike activity used a KGluconate based intracellular solution (in mM: 137.5 KGluconate, 2.5 KCl, 10 HEPES, 4 NaCl, 0.3 GTP, 4 ATP, 10 phosphocreatine, pH 7.5). Voltage-clamp recordings for IPSC measurements used a CeMeSO_4_ based intracellular solution (in mM: 114 CeMeSO_4_, 4 NaCl, 10 HEPES, 5 QX314, 0.3 GTP, 4 ATP, 10 phosphocreatine, pH 7.5). Alexa Fluor 594 (25 uM; Invitrogen) and Fluo-8 (250 uM;) were added to intracellular solution for calcium imaging experiments. In some experiments the following were added as indicated in the text: 10 µM CNQX or 5 µM NBQX, 50 µM D-AP5, 10 µM GABAzine were diluted from stock in the aCSF. All drugs were obtained from Tocris Biosciences, Inc. Intracellular recordings were made using a MultiClamp700B amplifier (Molecular Devices, Sunnyvale, CA) interfaced to a computer using a analog to digital converter (PCI-6259; National Instruments, Austin, TX) controlled by custom scripts in Igor Pro (www.wavemetrics.com; written by J.T.D.).

### Two-photon Imaging

All imaging experiments were carried out using a dual scan head raster scanning confocal microscope and control software developed by Prairie Systems (Middleton, WI) and incorporated into a BX51 upright microscope (Olympus America, Inc., Center Valley, PA). A x-y translation stage with integrated RIVETS system (Figure 4c) was used to position the tissue for imaging. Two-photon excitation was used with a Mai Tai pulsed Ti:Sapphire laser (Spectraphysics). Custom linescan patterns (Figure 5) were implemented using software generously provided by Bill Karsh (Applied Physics and Instrumentation Group, Janelia Farm Research Campus). All image analysis and presentation was performed using ImageJ (rsbweb.nih.gov/ij/), Matlab 2013a (www.mathworks.com) and plotted using IgorPro with the exception of Figure 6d which was produced using Vaa3D (penglab.janelia.org/proj/vaa3d/Vaa3D/About_Vaa3D.html).
